# Mental Health and Behavioral Symptoms Among Children Living in Democratic Republic of Congo, by Sex and Age

**DOI:** 10.51894/001c.164039

**Published:** 2026-07-31

**Authors:** Saishreya Kankanalapalli, Nivedhya Sanju, Micahel Boivin, Desire Tshala, Itziar Familiar-Lopez

**Affiliations:** 1 College of Osteopathic Medicine, Michigan State University

## 23

### Background

There is limited information on the distribution of mental health symptoms among children in rural, low-resource settings.

### Objectives

We aimed to describe the distribution of mental health and behavioral symptoms by age and sex among a sample of children living in rural Democratic Republic of Congo (DRC), using standardized measures.

### Methods

This analysis used the baseline assessments of a study of long-term mental health outcomes of an early child development intervention in Kahemba, DRC. We used the DSM-5-TR parent-rated level 1 cross-cutting measure for mental health symptoms, classifying participants as DSM-positive if they endorsed symptoms meeting the threshold for further clinical attention in at least one domain. The Strengths and Difficulties Questionnaire (SDQ) was used to assess emotional, conduct, hyperactivity, peer relationships, and prosocial behaviors. Means and standard deviations (SDs) were calculated for continuous variables, and frequencies and percentages were used for categorical variables. T-tests or chi-square tests were used to examine differences by sex and age, accordingly.

### Results

The sample included 186 children, of which 102 were females (55%), and the mean age was 7.6 years (range 3.8-12.4 years). Sixty percent of children had DSM-5 positive symptoms, and the most frequently reported domains were somatic (31%), repetitive (28%), and anxiety (27%) symptoms. The proportion of children meeting the DSM-5 cross-cutting threshold for any domain did not differ by sex. Psychosis (*p*=.002) and somatic symptoms (*p*=.01) were significantly higher among children 5 years and older. The mean total SDQ was within the normal range (10.8) as were the sub-scale scores, and no significant differences were observed by sex. SDQ hyperactivity and total score were slightly higher in children younger than 5 years, although not significant.

### Discussion

These findings indicate that a substantial proportion of children screened positive for mental health symptoms, with somatic, repetitive, and anxiety symptoms most frequently reported. Age-related differences in psychosis and somatic symptom domains suggest that certain symptom patterns may become more prominent in older children, underscoring the importance of age-sensitive mental health screening in this population.

